# Isokinetic Knee Strength and Multidirectional Jump-Landing Asymmetry in Professional Football Players After Anterior Cruciate Ligament Reconstruction: An Exploratory Cross-Sectional Study

**DOI:** 10.3390/jcm15218233

**Published:** 2026-10-22

**Authors:** Łukasz Oleksy, Anna Mika, Maciej Kuchciak, Miłosz Szczudło, Paulina Ciepiela, Maciej Daszkiewicz, Artur Stolarczyk, Olga Adamska, Paweł Reichert, Renata Kielnar

**Affiliations:** 1 Department of Orthopedics, Traumatology and Hand Surgery, Faculty of Medicine, Wroclaw Medical University, 50-556 Wroclaw, Poland; loleksy@oleksy-fizjoterapia.pl (Ł.O.); pawel.reichert@umw.edu.pl (P.R.); 2 Institute of Physical Culture Sciences, University of Szczecin, 71-065 Szczecin, Poland; 3 Oleksy Medical & Sport Sciences, 37-100 Łańcut, Poland; 4 Institute of Clinical Rehabilitation, University of Physical Culture in Kraków, 31-571 Kraków, Poland; 5 Faculty of Physical Culture Sciences, Collegium Medicum, University of Rzeszów, 35-959 Rzeszów, Poland; mkuchciak@ur.edu.pl (M.K.); mszczudlo@ur.edu.pl (M.S.); 6 Institute of Physiotherapy, Faculty of Health Sciences and Psychology, Collegium Medicum, University Center for Research and Development in Health Sciences, University of Rzeszów, 35-315 Rzeszów, Poland; pciepiela@ur.edu.pl (P.C.); kielnarrenata@o2.pl (R.K.); 7 Physiotherapy Research Laboratory, University Centre of Physiotherapy and Rehabilitation, Faculty of Physiotherapy, Wroclaw Medical University, 50-368 Wrocław, Poland; maciej.daszkiewicz@student.umw.edu.pl; 8 Department of Orthopaedics and Rehabilitation, Medical and Dentistry Faculty, Medical University of Warsaw, 02-091 Warsaw, Poland; artur.stolarczyk@wum.edu.pl; 9 Department of Ophthalmology, Faculty of Medicine, Collegium Medicum, Cardinal Stefan Wyszyński University in Warsaw, 01-815 Warsaw, Poland; o.adamska@uksw.edu.pl

**Keywords:** anterior cruciate ligament reconstruction, isokinetic strength, interlimb asymmetry, 3D force plate, multidirectional jumping, football

## Abstract

**Background/Objectives:** Isokinetic testing is widely used after anterior cruciate ligament reconstruction (ACLR), but isolated knee strength may not reflect function during braking, re-acceleration, and stabilization. This study examined relationships between isokinetic strength and vertical and multidirectional performance in professional football players after ACLR and compared asymmetry across functional domains. **Methods:** Eleven male professional players, 12–40 months post-ACLR and back in unrestricted training and competition, completed isokinetic concentric knee extensor and flexor strength testing, single-leg countermovement and drop jumps, and unilateral and bilateral forward and lateral jumps on 3D force-plates. Limb differences, strength–performance associations, bilateral contributions, and domain-specific asymmetry were assessed. **Results:** No operated–non-operated limb difference remained significant after Holm correction. Knee extensor strength was positively associated with drop-jump reactive strength index (ρ = 0.612), countermovement-jump height (ρ = 0.491), anterior–posterior braking impulse during forward hop-in (ρ = 0.382), and propulsive impulse during forward hop-out (ρ = 0.442); however, none of the eight prespecified strength–performance correlations was statistically significant either before or after Holm adjustment. Median asymmetry differed across domains, reaching 20.9% for temporal control and stabilization and 14.7% for directional braking, compared with 9.9% for strength and 6.2% for vertical performance. Fifty percent of players had a median multidirectional-task asymmetry ≥20%. The operated limb contributed 49.9–53.0% of the total output during bilateral tasks. **Conclusions:** The absence of significant group-level differences in isokinetic strength and vertical jump outcomes did not exclude asymmetries in directional braking and stabilization. Isokinetic, vertical, and multidirectional assessments provide complementary, non-interchangeable information after ACLR. Multidirectional testing may therefore provide additional information on movement strategies that is not captured by strength and vertical jump tests alone.

## 1. Introduction

Return to professional football following anterior cruciate ligament reconstruction (ACLR) is not equivalent to restoration of the preinjury level of function. The proportion of football players who resume training and competition is higher than the proportion who regain their previous level of sports performance [1]. Similarly, the timing of the first competitive match, the number of minutes played, or availability for training does not establish whether an athlete has recovered the capacity to generate force, rapidly reduce momentum, accept load on one limb, and control the limb in different directions [2,3]. The contemporary concept of return to performance therefore extends assessment beyond participation alone and includes the athlete’s current capacity to perform tasks corresponding to the demands of the sport [4]. In football, these demands include repeated acceleration and deceleration, vertical and horizontal propulsion, landing following forward or lateral displacement, rapid transition from load absorption to renewed propulsion, and stabilization. Assessment after return to competition should therefore consider both muscular capacity and its use during dynamic tasks.

Knee extensor and flexor strength constitutes a fundamental component of this functional profile. The knee extensors generate the extension moment required during propulsion and control knee flexion during load acceptance, whereas the knee flexors contribute to dynamic joint control and may be particularly relevant following reconstruction with a hamstring tendon autograft [5]. Isokinetic dynamometry provides a standardized assessment of peak torque at a predefined angular velocity, while normalization to body mass enables comparison between athletes of different body sizes [6,7,8,9]. Strength recovery after ACLR is not uniform. A meta-analysis including 232 studies and more than 34,000 participants showed that the greatest improvement occurs during the early postoperative period, slows at approximately 12–18 months, and may remain incomplete thereafter [6]. Deficits relative to healthy individuals may also be greater than those identified when the contralateral limb is used as the sole reference. A high level of inter-limb symmetry may coexist with reduced performance of both limbs; therefore, interpretation should consider the relative torque of each limb, testing velocity, performance level, graft type, and time since reconstruction [5,6,9].

The isolated capacity to generate joint torque is not equivalent to performance during a dynamic task. Isokinetic testing assesses controlled movement at a single joint, whereas jumping and landing require coordinated contributions from the hip, knee, and ankle, trunk control, rapid force development, and adjustment of the movement strategy to the direction and phase of the task [6,9]. Knee strength may be associated with better functional performance but explains only part of the outcome. Crotty et al. [10] reported that knee extensor strength explained approximately 29% of the variance in single-leg drop jump (SLDJ) height and 33% of the variance in reactive strength index (RSI). In professional football players after ACLR, lower knee extensor strength was associated with lower SLDJ height, RSI, and power, although RSI-based classification differentiated more jump variables than classification based solely on knee extensor strength [11]. Associations between isokinetic strength and multidirectional hop performance also vary with the examined muscle group and testing velocity [12,13]. Conversely, acceptable hop performance may coexist with insufficient knee extensor strength, and hop outcomes do not allow for accurate estimation of an individual athlete’s strength [14,15]. Final jump height or distance may be maintained through altered contributions from the hip and ankle, trunk positioning, or landing strategy [16,17]. Comparable hop distance has been reported after ACLR despite reduced knee flexion and altered joint moments [17], while larger deficits may be identified in single-leg vertical jumps than in tests based only on horizontal distance [18]. Strength and jump outcomes should therefore be considered complementary rather than interchangeable.

The single-leg countermovement jump (SLCMJ) and SLDJ provide a bridge between isolated strength assessment and directional tasks. The SLCMJ reflects vertical force generation during unilateral propulsion, whereas the SLDJ additionally requires rapid load acceptance and transition to renewed propulsion. Jump height, RSI, contact time, and braking and propulsive impulses describe different aspects of performance [19,20,21,22,23]. Phase-specific differences have been reported in professional football players after ACLR even when the final jump outcome was acceptable [20,24]. Vertical assessment should therefore consider both the achieved outcome and the strategy used to obtain it, but it does not fully represent forward and lateral movement demands.

The relationship between strength and function may also vary across movement phases. Directional tasks can separate impulse generation before take-off from load acceptance, braking, re-propulsion, and stabilization after landing. Knee extensor strength may support load absorption, as illustrated by its association with knee joint moment during landing [25]. However, external ground reaction force impulse is not a direct measure of knee joint moment because it also reflects approach velocity, trunk position, and contributions from the hip and ankle.

Task direction introduces additional demands. Lateral hops require control in the frontal and transverse planes [26], while landing direction modifies stability outcomes in the vertical, anterior–posterior (AP), and medial–lateral (ML) directions [27,28]. Time to stabilization integrates lower-limb and trunk control and may remain altered after ACLR [29,30], but it should not be interpreted as a direct measure of knee strength or as a stand-alone predictor of subsequent injury. Associations between isokinetic strength and stabilization may therefore be weaker and more heterogeneous than associations with vertical propulsion.

The number of limbs involved may further influence task performance. During bilateral jumping, impulse generation and absorption can be distributed between the limbs, allowing the whole-body outcome to remain acceptable despite unequal contributions [24,31,32]. Unilateral tasks reduce this opportunity but increase the demands placed on balance, pelvic and trunk control, and landing organization [33,34]. Comparisons between bilateral and unilateral conditions may therefore indicate whether compensation modifies the relationship between strength and task performance, but they do not establish the universal superiority of either task type.

Previous studies have focused primarily on relationships between knee strength and jump height, RSI, or single-leg hop distance [10,11,12,13,14,15]. Less is known about whether isolated knee extensor and flexor strength is associated with directional impulse generation, braking, rapid re-propulsion, and stabilization measured using three-dimensional force platforms. This knowledge gap is relevant in professional football players who have returned to full training and competition, because satisfactory overall outcomes may coexist with differences that are specific to a movement phase or direction. The primary aim of this study was therefore to examine associations between isokinetic knee extensor and flexor strength and the vertical and directional use of the lower limb during acceleration, braking, re-propulsion, and stabilization in professional football players after ACLR. The secondary aim was to compare the operated and non-operated limbs and determine whether relationships between strength and task performance differed between unilateral and bilateral tasks. We hypothesized that: (1) the operated limb would demonstrate lower relative knee-extensor and knee-flexor peak torque at 60°/s, SLCMJ height, and SLDJ RSI than the non-operated limb; (2) operated-limb knee-extensor and knee-flexor strength at 60°/s would be positively associated with SLCMJ height, SLDJ RSI, AP braking impulse during the unilateral forward hop-in, and AP propulsive impulse during the unilateral forward hop-out; (3) the corresponding strength–performance associations would be stronger for unilateral than bilateral task variants; and (4) median absolute asymmetry would be greater for directional braking and temporal control and stabilization than for isokinetic strength and vertical performance. Inter-limb asymmetry was treated as a descriptive, task-specific difference and not as evidence of clinical abnormality or a consequence of ACLR.

## 2. Materials and Methods

### 2.1. Study Participants

Twelve male professional football players who had undergone primary anterior cruciate ligament reconstruction (ACLR) were initially enrolled. The final analytical sample comprised 11 players aged 19–30 years. In the final sample, body mass was 64.2 ± 8.5 kg and height was 1.75 ± 0.12 m (mean ± SD). The operated side was right in six players and left in five; limb dominance was not recorded. Time since surgery ranged from 12 to 40 months. All players had sustained a first complete ACL rupture accompanied by a meniscal injury requiring surgical repair. ACL reconstruction was performed using an autologous hamstring tendon graft. Detailed information regarding meniscal tear location, tear pattern, and surgical repair technique was not consistently available from the medical records. At the time of testing, all players had completed the return-to-sport (RTS) process according to the criteria applied by their clubs’ medical and strength and conditioning staff and had resumed full participation in team training and official professional matches. None of the players reported symptoms related to the reconstructed knee that limited their current participation in training or competition. The specific tests, thresholds, dates, and individual results used by the clubs for RTS clearance were not available in a standardized verifiable form and were not used as a reference standard in the present study.

All participants provided written informed consent before data collection. The study protocol was approved by the Ethics Committee of the Regional Medical Chamber in Kraków (No. 35/KBL/OIL/2024) and was conducted in accordance with the Declaration of Helsinki (1964) and its subsequent amendments.

### 2.2. Study Design

This was an observational, cross-sectional study. Each participant attended a single laboratory testing session, and all measurements were performed by the same team of experienced researchers. Body mass and height were recorded before testing, after which the players completed a standardized 10 min warm-up.

The assessment battery comprised three complementary components. First, the isolated strength of the knee extensors and flexors was measured under isokinetic conditions. Second, single-leg vertical jump tests were used to assess vertical propulsion and the rapid transition from landing to subsequent take-off. Third, forward and lateral jump–landing tasks were performed to evaluate propulsion, braking, re-propulsion, and post-landing stabilization. Together, these assessments characterized isolated muscular capacity and its use during dynamic tasks of increasing complexity. Both lower limbs were included in the analysis. Each result was assigned to the operated limb (O) or the non-operated limb (NO) according to verified clinical records.

### 2.3. Eligibility Criteria and Quality Control

The inclusion criteria were male sex, professional participation in football, a first complete ACL rupture treated surgically, reconstruction using an autologous hamstring tendon graft, completion of at least 12 months of postoperative rehabilitation and training, successful completion of the RTS process, full participation in professional training and competition, and the absence of symptoms related to the reconstructed knee that limited current sports participation.

Before statistical analysis, all recordings were assessed for completeness, visible signal artifacts, and correct identification of movement phases. One player was excluded from the entire analysis because the recorded data were of insufficient quality to permit reliable identification of movement phases and comparison between limbs. Although the final analytical sample comprised 11 players, the number of valid observations varied between outcomes because of outcome-specific data availability. Exact sample sizes are therefore reported for the corresponding analyses.

### 2.4. Experimental Procedures

#### 2.4.1. Isokinetic Strength Assessment

Isokinetic knee strength was assessed using a Biodex S4 dynamometer (Biodex Medical Systems, Shirley, NY, USA). Both limbs were tested during concentric knee extension and flexion at angular velocities of 60°/s, 180°/s, and 300°/s. At each angular velocity, one recorded set was completed with each limb, comprising 5, 10, and 15 maximal repetitions at 60°/s, 180°/s, and 300°/s, respectively. Peak torque was defined as the highest torque recorded within the corresponding set and was normalized to body mass and expressed in Nm/kg. The non-operated limb was tested first, and a recovery interval of 30 s was provided between successive sets.

#### 2.4.2. Vertical Jump Assessment

Vertical jump performance was recorded at a sampling frequency of 1000 Hz using two Hawkin Dynamics force platforms (Hawkin Dynamics, Westbrook, ME, USA). All tasks were performed in sports footwear, with the players keeping their hands on their hips from movement initiation until completion of the final landing. The tests were conducted in the following fixed order: bilateral countermovement jump (CMJ), single-leg countermovement jump (SLCMJ), bilateral drop jump (DJ), and single-leg drop jump (SLDJ). Three valid trials were completed for each bilateral task and with each limb during the unilateral tasks. During the SLCMJ and SLDJ, the non-operated limb was tested first, and successive trials alternated between the non-operated and operated limbs. A trial was considered valid when the required take-off and landing were completed using the designated limb or limbs without loss of balance, additional ground contact, or removal of the hands from the hips. Invalid attempts were repeated until three valid trials were obtained. Recovery intervals of 15 s between trials and 60 s between different jump tasks were provided. For each task and each limb, the mean of the three valid trials was used for statistical analysis.

The primary vertical tasks were the SLCMJ and SLDJ. During the SLCMJ, the player began in a single-leg standing position and performed a maximal vertical jump using a self-selected countermovement. Jump height and net vertical propulsive impulse were retained for analysis. During the SLDJ, the player stepped from a 24 cm box, landed on one limb, and immediately performed a maximal vertical jump using the same limb. Jump height, reactive strength index (RSI), ground contact time, and net vertical impulses during the braking and propulsive phases were analyzed. RSI was calculated as jump height divided by ground contact time. All impulse variables were normalized to body mass and expressed in N·s·kg^−1^. The CMJ and DJ were included as supplementary assessments. For the CMJ, jump height and the operated limb’s contribution to total propulsive impulse were retained. For the DJ, RSI and the operated limb’s contribution to total braking impulse were analyzed.

#### 2.4.3. Directional Jump–Landing Tasks

Three-dimensional ground reaction force data were recorded at a sampling frequency of 480 Hz using two synchronized Launch Pad force platforms (Axioforce, Lawrence, KS, USA). The system recorded the vertical (V; Fz), anterior–posterior (AP; Fy), and medial–lateral (ML; Fx) components of the ground reaction force. During bilateral tasks, each foot was positioned on a separate platform, allowing for the contribution of each limb to be determined. Unilateral tasks were performed separately with the operated and non-operated limbs. For each directional task, three valid recorded trials were completed with each limb. The non-operated limb was tested first, and successive trials were alternated between limbs. A 15 s recovery interval was provided between trials and a 60 s recovery interval between directional tasks. For tasks initiated outside the force platforms, the starting distance from the platform was standardized. Floor markers were placed on all four sides of the force-platform setup to indicate the required starting position and ensure a consistent take-off distance across directions, limbs, and repeated trials. Although the starting distance was standardized, take-off intensity was not independently quantified or externally controlled. A trial was considered valid when the player completed the required take-off and landing using the designated limb, landed fully on the force platform, and maintained balance without contralateral-limb contact or an additional step. Invalid trials were repeated. The mean of all valid trials was used for statistical analysis.

The directional testing protocol comprised five tasks:

1. Forward hop-in: a forward jump from the floor onto the force platforms, followed by landing and stabilization. This task was performed bilaterally and unilaterally.
2. Forward hop-out: a forward jump initiated from the force platforms and performed under bilateral and unilateral conditions. This task was used to assess the propulsive phase preceding take-off.
3. Forward hop-in–out: a combined forward task in which the player landed on the force platforms and immediately performed a second take-off to land beyond them. Bilateral and unilateral variants were completed.
4. Lateral hop-in: a unilateral lateral jump from the floor onto the force platform, followed by stabilization.
5. Lateral hop-out: a unilateral lateral jump initiated from the force platform and used to assess propulsion in the ML direction.

### 2.5. Processing of Directional Force-Platform Signals

Raw force signals from the directional tasks were processed using custom scripts written in Python version 3.12.13 (Python Software Foundation; Beaverton, OR, USA). The force platforms were used with the manufacturer-provided calibration for all three force axes. Quiet standing was not used to calibrate AP or ML force sensitivity. Instead, it was used to verify vertical loading and derive a common scaling factor, calculated as verified body weight in newtons divided by the mean total raw Fz during quiet standing. The same scaling factor was applied to Fx, Fy, and Fz. Mean quiet-standing offsets were subsequently subtracted from the AP and ML signals. No additional independent calibration of the AP or ML channels was performed. A fourth-order, zero-phase Butterworth low-pass filter with a cutoff frequency of 50 Hz was applied to all force channels. Initial contact and take-off were identified when vertical force increased above or decreased below 0.20 body weight (BW), respectively. The 0.20 BW threshold was selected a priori as a body-mass-normalized operational criterion to distinguish true platform loading and unloading from baseline noise and low-amplitude force fluctuations. The same threshold was applied consistently across all participants and directional tasks. For bilateral tasks, initial contact and take-off were identified from the summed vertical force recorded by both platforms, whereas limb-specific impulses were calculated separately from the force recorded by each platform. The AP and ML signals were corrected relative to their quiet-standing baseline values. Signed AP and ML signals were used to determine movement direction and identify the boundaries of individual phases. AP and ML impulses were calculated by integrating the absolute force values within the corresponding phase. The resulting AP and ML values therefore represent absolute impulses reflecting the cumulative magnitude of directional loading within a predefined task phase. They are not signed net impulses and do not indicate the direction of the net momentum change. This approach limited the cancellation of forces acting in opposite directions and reduced the influence of force-axis polarity and operated-limb laterality on the results. Accordingly, the terms “braking” and “propulsion” refer to the predefined task phases rather than to the sign of the integrated force. Throughout phase detection, a force change was considered sustained when the relevant criterion was maintained for at least 15 consecutive samples, corresponding to 31.25 ms at 480 Hz. For tasks ending with landing on the force platform, the braking phase began at initial contact and ended when Fz first decreased and remained at or below 1 BW after the vertical force peak. This force-based boundary was used as an operational criterion to delimit the load-acceptance phase. It should not be interpreted as the instant of zero center-of-mass velocity or as the exact end of AP or ML deceleration. For tasks initiated on the force platform, the propulsive phase began with the first sustained increase in force above 5% BW in the primary direction of movement and ended at take-off. Take-off was identified when Fz decreased below 0.20 BW. For the combined forward hop-in–out task, the platform contact period was divided into a braking phase and a subsequent propulsive phase. The transition was identified using one of two predefined rules: (1) a sustained reversal in the direction of the AP force signal or (2) the minimum absolute AP force within the contact period when a clear reversal was absent. These rules were applied in 25/31 and 6/31 evaluable recordings, respectively. Total contact time was calculated from initial contact to subsequent take-off. Time to stabilization (TTS), used as a force-platform measure of post-landing dynamic stability [35,36], was defined as the interval from initial contact to the beginning of the first continuous 0.5 s period during which force in the primary task axis remained within ±5% BW of the quiet-standing reference value. No additional moving-window smoothing was applied after low-pass filtering. Any excursion beyond the ±5% BW limits invalidated the candidate interval, and the search continued until the first continuous period meeting the criterion was identified. The 0.5 s requirement was used to identify a sustained return toward the quiet-standing reference rather than a transient threshold crossing and was applied consistently to all trials.

### 2.6. Outcome Variables

The primary explanatory variables were the relative peak torque values of the knee extensors and flexors of the operated limb measured at 60°/s. Four outcomes were selected a priori to represent different aspects of dynamic strength use:

1. SLCMJ height, representing vertical propulsion;
2. SLDJ RSI, representing the rapid transition from landing to subsequent take-off;
3. AP braking impulse during the unilateral forward hop-in;
4. AP propulsive impulse during the unilateral forward hop-out.

Secondary analyses included the remaining SLCMJ and SLDJ outcomes; TTS after the unilateral forward hop-in; braking impulse, subsequent propulsive impulse, and contact time during the combined unilateral forward hop-in–out; braking impulse and TTS during the lateral hop-in; and propulsive impulse during the lateral hop-out.

All impulse variables were normalized to body mass and expressed in N·s·kg^−1^. For bilateral tasks, both the combined result of the two limbs and the relative contribution of the operated limb were calculated:

$$Operated\;limb\;contribution=\frac{O}{O+NO}$$

where *O* represents the value obtained for the operated limb and *NO* represents the value obtained for the non-operated limb. A value of 0.50 indicated equal measured contributions from the two limbs.

### 2.7. Statistical Analysis

Because of the small sample size, continuous variables were summarized as the mean and standard deviation or as the median and interquartile range [Q1–Q3], depending on their distribution. Categorical variables are presented as counts and percentages.

For each variable, the paired difference was calculated as Δ = O − NO. The normality of paired differences was assessed using the Shapiro–Wilk test, and potential outliers were identified using the modified median absolute deviation (MAD) method. The operated and non-operated limbs were compared using a paired-samples t-test when the distribution of paired differences was approximately normal and contained no outliers. Otherwise, the Wilcoxon signed-rank test was used. The paired difference, its bootstrap 95% confidence interval (CI), and the effect size were reported. Effect sizes were expressed as the small-sample bias-corrected Hedges’ g_z for paired t-tests and the rank-biserial correlation coefficient (r_rb) for Wilcoxon tests.

Directional inter-limb asymmetry was calculated for each variable as follows [37]:

$$Directional\;asymmetry(\%)=100\times \frac{O-NO}{max(|O|,|NO|)}$$

where *O* denotes the value obtained for the operated limb and *NO* denotes the value obtained for the non-operated limb. Positive values indicated a higher result for the operated limb, whereas negative values indicated a higher result for the non-operated limb. The absolute value of asymmetry was used to describe the magnitude of the inter-limb difference irrespective of direction.

Associations between knee strength and dynamic task performance were assessed using Spearman’s rank correlation coefficient (ρ). In the primary analysis, the relative peak torque of the operated-limb knee extensors and flexors at 60°/s was correlated with four outcomes from the same limb: SLCMJ height, SLDJ RSI, AP braking impulse during the unilateral forward hop-in, and AP propulsive impulse during the unilateral forward hop-out. These eight primary correlations were divided into two families of four correlations: one for the knee extensors and one for the knee flexors. Permutation-based *p*-values and bootstrap 95% CIs were calculated for the correlation coefficients. Additional analyses included the remaining vertical and directional outcomes, associations between strength asymmetry and task-performance asymmetry, and isokinetic outcomes obtained at 180°/s and 300°/s. The strength of corresponding correlations for vertical and directional outcomes was compared using a participant-level paired bootstrap, in which the same resampled players contributed to both correlations. For bilateral tasks, the operated-limb contribution to the combined result was calculated as O/(O + NO). These values were compared with 0.50, representing equal contributions from the two limbs, using a one-sample t-test or Wilcoxon signed-rank test, according to the distribution and outlier criteria described above. The four comparisons reported for the bilateral directional tasks formed one family for Holm adjustment. The analyses also examined whether strength asymmetry was associated with the operated-limb contribution and whether the mean strength of the two limbs was associated with the combined task result. Corresponding associations obtained in unilateral and bilateral variants of the same task were compared using a participant-level paired bootstrap. To compare the magnitude of asymmetry across different components of function, the median absolute asymmetry was calculated for each player within five domains: isokinetic strength, vertical outcomes, directional braking, propulsion and re-acceleration, and temporal control and stabilization. The domains were compared using the Friedman test, with Kendall’s coefficient of concordance (W) reported as the effect size. Post hoc comparisons were performed using Wilcoxon signed-rank tests. The ten pairwise comparisons among the five domains formed one family for Holm adjustment. At the individual level, the proportion of players with an absolute asymmetry of at least 20% was determined. The analysis considered both the presence of at least one outcome exceeding this threshold and a median absolute asymmetry of at least 20% across the full set of directional tasks. The 20% value was selected as a conservative operational threshold, corresponding to twice the commonly used 10% asymmetry level conceptually associated with a 90% limb symmetry index. Because this threshold has not been validated for the force-impulse or stabilization measures examined, it was used descriptively and should not be interpreted as a clinical cut-off. The frequency of threshold exceedance in knee strength and the primary vertical outcomes was compared with that observed in the directional tasks using the exact McNemar test. The exact McNemar comparisons performed at the 20% threshold formed one family for Holm adjustment. For operated–non-operated limb comparisons, Holm adjustment was applied separately to four families of outcomes: isokinetic strength, vertical jumps, forward directional tasks, and lateral directional tasks. The primary strength–performance correlations, post hoc comparisons between functional domains, comparisons of operated-limb contribution with 0.50, and threshold-based McNemar comparisons were adjusted within the separate families defined above. All tests were two-sided, and statistical significance was set at α = 0.05. A sensitivity analysis was performed for the final sample size. With *n* = 11, a two-sided α = 0.05, and 80% power, the minimum detectable effects were approximately |r| = 0.72 for correlations and d_z = 0.94 for paired comparisons. These values should be considered approximate because they are based on the parametric equivalents of the statistical tests used in the study. The primary analyses were performed using STATISTICA 13.0 (TIBCO Software Inc., Palo Alto, CA, USA). Modified MAD outlier detection, permutation-based *p*-values, bootstrap 95% CIs, effect-size estimates, paired-bootstrap comparisons of correlation coefficients, exact McNemar tests, and Holm adjustments were implemented in Python 3.12.13 using the NumPy and SciPy libraries.

## 3. Results

### 3.1. Comparison Between the Operated and Non-Operated Limbs

At the group level, no statistically significant differences were found between the operated and non-operated limbs. This applied to knee extensor and flexor strength, vertical jump height and reactive performance, and braking, propulsion, re-propulsion, and stabilization outcomes during forward and lateral tasks. The lowest unadjusted *p*-values were observed for contact time during the unilateral forward hop-in–out (*p* = 0.037; Holm-adjusted *p* = 0.223), AP re-propulsive impulse during the same task (*p* = 0.061; Holm-adjusted *p* = 0.306), and knee flexor strength at 60°/s (*p* = 0.091; Holm-adjusted *p* = 0.549). None of these differences remained significant after Holm adjustment (Table 1). Additional operated–non-operated limb comparisons for knee-extensor and knee-flexor PT/BW at 180°/s and 300°/s are presented in Appendix A.1 (Table A1). Accordingly, no consistent group-level pattern indicated systematically poorer performance of the operated limb. However, the absence of a systematic difference at the group level did not indicate limb equivalence in individual players. Both the magnitude and direction of interlimb differences varied considerably between players.

### 3.2. Associations Between Isokinetic Strength and Dynamic Task Outcomes

In the primary analyses, neither operated-limb knee extensor nor flexor strength at 60°/s was significantly associated with the selected dynamic task outcomes after Holm adjustment. All primary correlation coefficients were positive, indicating that greater strength generally tended to accompany higher values of the analyzed outcomes, but the uncertainty around these estimates was substantial.

The strongest observed association was between knee extensor strength and SLDJ RSI (ρ = 0.612; 95% CI, −0.259 to 0.963; *p* = 0.068; Holm-adjusted *p* = 0.271). Players with greater knee extensor strength therefore tended to demonstrate better reactive performance during the SLDJ, although this relationship was not statistically significant after adjustment. The association between knee extensor strength and SLCMJ height was moderate (ρ = 0.491; 95% CI, −0.246 to 0.923; *p* = 0.158; Holm-adjusted *p* = 0.474). The corresponding correlations with AP braking impulse during the forward hop-in and AP propulsive impulse during the forward hop-out were ρ = 0.382 and ρ = 0.442, respectively. Neither association was statistically significant after Holm adjustment.

For the knee flexors, the strongest correlation was observed with AP braking impulse during the forward hop-in (ρ = 0.515; *p* = 0.134; Holm-adjusted *p* = 0.537). Correlations with SLCMJ height, SLDJ RSI, and AP propulsive impulse during the forward hop-out were ρ = 0.152, ρ = 0.188, and ρ = 0.382, respectively, and none remained significant after correction for multiple comparisons (Table 2). The data did not support stronger associations of knee strength with vertical tasks than with directional tasks. Additional analyses using strength measured at 180°/s and 300°/s also showed no significant associations. Furthermore, greater strength asymmetry was not systematically accompanied by greater asymmetry during the dynamic tasks. The complete results of the additional strength–performance correlation analyses are presented in Appendix A.2 (Table A2). Comparisons of the corresponding strength–performance correlations between unilateral and bilateral variants of the directional tasks are presented in Appendix A.3 (Table A3).

### 3.3. Magnitude of Asymmetry Across Functional Domains

To determine which components of task performance showed the greatest inter-limb differences, absolute asymmetry was summarized across five domains: isokinetic strength, vertical jump outcomes, directional braking, propulsion and re-acceleration, and temporal control and stabilization. Absolute values were used so that the magnitude of asymmetry was evaluated irrespective of which limb produced the higher result.

The smallest median asymmetry was observed for vertical jump outcomes (6.2%). Asymmetry was slightly greater for propulsion and re-acceleration (7.3%) and isokinetic strength (9.9%), increased to 14.7% for directional braking, and reached its highest value for temporal control and stabilization after landing (20.9%). Differences among the five domains were statistically significant (χ^2^(4) = 18.80; *p* < 0.001; Kendall’s W = 0.522).

Temporal control and stabilization showed 11.1 percentage points greater asymmetry than isokinetic strength and 8.3 percentage points greater asymmetry than propulsion and re-acceleration. Directional braking also showed 10.5 percentage points greater asymmetry than vertical jump outcomes. All three post hoc differences were statistically significant (Holm-adjusted *p* = 0.039); no other between-domain comparison was significant (Table 3).

### 3.4. Asymmetry During Complex Directional Tasks

The largest individual inter-limb differences were observed during directional braking and recovery of stability after landing. At the 20% threshold, 10/10 assessed players (100%) demonstrated at least one directional braking, re-propulsion, or stabilization outcome at or above 20%. In 5/10 players (50%), the median absolute asymmetry across the complete set of directional tasks was also at least 20%. In 8/10 players (80%), at least one asymmetry above 20% was identified during a directional task despite no corresponding threshold exceedance in knee strength or the primary vertical jump outcomes. The opposite pattern was observed in 0/10 players. This paired difference was statistically significant (Holm-adjusted *p* = 0.016).

To determine whether this finding reflected only the larger number of variables available for the directional tasks, the comparison was repeated using two outcomes from each set. Even after equalizing the number of variables, 7/10 players (70%) showed an asymmetry above 20% exclusively during directional braking or re-propulsion, whereas the opposite pattern, involving an asymmetry exclusively in the selected vertical jump-height outcomes, was observed in 0/10 players. This difference also remained statistically significant (Holm-adjusted *p* = 0.016) (Table 4).

### 3.5. Contribution of the Operated Limb During Bilateral Tasks

Across all bilateral directional tasks, the mean contribution of the operated limb was close to 50%, ranging from 49.9% to 53.0%. None of the values differed significantly from equal contribution by the two limbs after adjustment for multiple comparisons (Table 5). Therefore, no systematic reduction in operated-limb contribution was identified at the group level during bilateral directional tasks.

A similar distribution was observed during the bilateral vertical jumps. The operated limb contributed a mean of 49.3% of the total propulsive impulse during the CMJ and 51.2% of the total braking impulse during the DJ.

Strength asymmetry was not consistently associated with lower operated-limb contribution during bilateral tasks. The association between knee strength and vertical or directional task performance was also not demonstrably stronger in unilateral than in bilateral conditions. Thus, within the present sample, the opportunity to use both limbs did not systematically modify the relationship between knee strength and dynamic task performance.

## 4. Discussion

The aim of the present study was to determine the extent to which isolated knee extensor and flexor strength was reflected in lower-limb performance during vertical and directional dynamic tasks in professional football players after ACLR. The principal finding was that the functional profile depended on the task examined. At the group level, the operated limb did not demonstrate systematically poorer isokinetic strength, SLCMJ or SLDJ performance, or forward and lateral task outcomes than the non-operated limb. Despite this group-level result, absolute knee-extensor PT/BW asymmetry at 60°/s was at least 10% in 6 of 11 players (54.5%). This descriptive threshold-based finding highlights individual variability but should not be interpreted as failure to meet the overall RTS criteria, because a single strength threshold cannot reproduce the multidimensional return-to-sport decisions made by the clubs. Individual analysis, however, revealed substantial inter-limb asymmetries in specific movement phases, particularly during directional braking and the recovery of stability after landing. Knee strength was positively associated with several dynamic outcomes, but none of the primary correlations remained statistically significant after adjustment for multiple comparisons. These findings indicate that isokinetic strength represents an important component of dynamic performance but does not fully characterize how a player absorbs load, re-propels the body, or regains stability. The hypotheses were therefore only partly supported. The operated limb did not perform systematically worse across the assessed domains, and the positive direction of the estimates involving knee extensor strength was consistent with the hypothesized relationships. The data also did not demonstrate that strength–performance relationships were consistently stronger during unilateral than bilateral tasks. The findings most clearly supported the hypothesis that isokinetic strength and complex dynamic tasks describe partly distinct components of lower-limb function. Moreover, absolute asymmetry describes the magnitude of an inter-limb difference but does not identify which limb performed more favorably. A higher value has different implications for jump height, TTS, and braking impulse. Without preinjury data, a healthy reference group, and standardized approach intensity, the observed differences cannot uniformly be interpreted as deficits of the operated limb.

The absence of systematic differences between the operated and non-operated limbs should not be interpreted as evidence of complete functional recovery. Mean knee extensor peak torque normalized to body mass was 2.9 Nm/kg in the operated limb and 3.0 Nm/kg in the non-operated limb, while the corresponding knee flexor values were 1.7 and 1.8 Nm/kg. Median absolute asymmetry was 13.4% for the knee extensors and 7.8% for the knee flexors. Because both the magnitude and direction of interlimb differences varied among players, opposing individual patterns may have offset one another in the group-level analysis. A similar discrepancy between group-level results and individual strength and jump-kinetic profiles has been reported in elite female football players [32]. Group means should therefore not be used to infer that both limbs performed similarly in every player. Interpretation of inter-limb comparisons is further limited by the fact that the non-operated limb does not necessarily provide a normal reference. Girdwood et al. [6] showed that strength deficits relative to healthy controls may be greater than those suggested by comparison with the contralateral limb alone. Wellsandt et al. [38] similarly demonstrated that the limb symmetry index may overestimate recovery when performance of the non-operated limb is also reduced. Moreover, comparable outcomes between limbs during a hop test do not establish that an adequate absolute performance level or the remaining return-to-sport criteria have been achieved [39,40]. Strength should therefore be interpreted using the relative torque of each limb, the inter-limb difference, and reference values appropriate for sex, sport, performance level, and testing velocity [9]. Because the present study did not include preinjury data or a healthy control group, it was not possible to fully determine whether the observed values differed from preinjury values or from those expected in healthy professional football players.

The clearest strength–performance relationships were observed between knee extensor strength and unilateral vertical jump outcomes. Knee extensor strength was positively correlated with SLCMJ height (ρ = 0.49) and SLDJ RSI (ρ = 0.61), indicating that players with greater extensor strength generally jumped higher and achieved a more favorable ratio of jump height to ground contact time. The direction of these associations agrees with the results of Crotty et al. [10], who reported that knee extensor strength explained 29% of the variance in SLDJ height and 33% of the variance in RSI. Maestroni et al. [11] likewise showed in professional football players after ACLR that lower knee extensor strength was accompanied by lower SLDJ height, RSI, and power. Nevertheless, both studies indicate that knee extensor strength explains only part of jump performance. In the study by Crotty et al. [10], approximately two-thirds of the variance in RSI and an even greater proportion of the variance in contact time were not explained by knee extensor strength. Maestroni et al. [11] found that grouping players according to RSI differentiated more kinetic variables than grouping them solely according to knee extensor strength. Taken together, the present estimates and previous findings suggest that isokinetic strength and vertical jump performance may be related, but the magnitude and consistency of this relationship remain uncertain. These measures should be regarded as complementary rather than interchangeable, and the observed associations require confirmation in larger samples.

Isolated knee extensor strength may support vertical propulsion and reactive performance, but SLDJ execution also depends on rapid load acceptance, muscle-tendon unit properties, ground contact time, and an efficient transition from braking to subsequent propulsion. Isokinetic dynamometry therefore cannot replace direct assessment of jump performance. This interpretation is supported by Barfod et al. [14] and Turck et al. [15], who showed that acceptable hop performance may coexist with insufficient knee extensor strength and that hop outcomes do not estimate an individual athlete’s strength with sufficient accuracy. In the current study the associations of knee flexor strength with SLCMJ height and SLDJ RSI were weak (ρ = 0.15 and ρ = 0.19, respectively). The strongest flexor relationship was observed for AP braking impulse during the unilateral forward hop onto the platform (ρ = 0.52). Although this association was not statistically significant, it may indicate a possible task-specific relationship that requires confirmation in a larger sample. Aizawa et al. [12] reported moderate associations between knee flexor strength and hop distance in different directions, whereas Sueyoshi et al. [13] identified clearer relationships for knee flexor work measured at a higher angular velocity. All players in the present study had undergone reconstruction using a hamstring tendon autograft, which may have further contributed to individual variability in knee flexor capacity [5]. Therefore, knee flexor strength should be considered a distinct component of the functional profile rather than solely as part of the hamstring-to-quadriceps ratio. However, a hamstring-to-quadriceps ratio within the expected range may coexist with insufficient absolute strength in both muscle groups. Moderate positive but statistically non-significant correlations were also observed between knee extensor strength and forward-task outcomes. Higher extensor-strength values tended to coexist with greater AP braking impulse during the unilateral forward hop onto the platform (ρ = 0.38) and greater AP propulsive impulse during the unilateral forward hop from the platform (ρ = 0.44). These findings suggest that the knee extensors may contribute both to generating the impulse required for forward displacement and to controlling the limb during load acceptance. Oberländer et al. [25] similarly reported that the weaker limb after ACLR generated a lower knee joint moment during landing and that knee extensor strength was associated with peak knee joint moment. Their finding provides a mechanistic rationale for the role of the knee extensors in load absorption, but it is not a direct equivalent of the present measurements. A knee joint moment describes the contribution of a specific joint, whereas the impulse of the external ground reaction force reflects the net effect of the entire movement system. A similar impulse may therefore be produced through different contributions from the knee, hip, ankle, and trunk. Braking impulse must also be interpreted within the context of the complete task. In the present analysis, a greater absolute-force integral represents a greater cumulative magnitude of AP loading within the predefined phase. It does not represent a signed net impulse or directly quantify the net change in momentum. It may indicate the capacity to control a more dynamic landing, but it may also result from a higher velocity before contact. Conversely, a smaller impulse may reflect lower cumulative directional loading or a less dynamic execution strategy. Braking impulse should therefore be interpreted together with the preceding propulsion, approach intensity, ground contact time, and time required to regain stability. Although the starting distance was standardized, take-off intensity was not independently quantified or externally controlled. Consequently, differences in impulse magnitude may partly reflect individual variation in task execution strategy. The absence of a strong correlation with knee strength does not imply that strength is unimportant for braking; rather, it indicates that the outcome may depend on multiple factors, including muscular capacity and the movement strategy used to complete the task. The present data did not demonstrate that knee extensor strength was more strongly associated with vertical than directional performance, and the observed relationships require confirmation in larger samples.

The most distinct difference among the assessed functional domains concerned the magnitude of inter-limb asymmetry. Median absolute asymmetry was lowest for the primary vertical jump outcomes (6.2%) and for propulsion and re-acceleration variables (7.3%). It was 9.9% for isokinetic strength, increased to 14.7% for directional braking, and was greatest for temporal control and stabilization (20.9%). The between-domain difference was statistically significant. Directional braking showed greater asymmetry than vertical jump outcomes, while temporal control and stabilization showed greater asymmetry than strength and propulsion. Thus, relatively small inter-limb differences in strength and vertical jump outcomes did not necessarily coincide with similarly small differences in load-acceptance or stabilization measures. This finding extends previous observations of discordance between the final jump outcome and the strategy used to achieve it. Davies et al. [16] emphasized that hop distance may be preserved through redistribution of the contribution of individual joints. Trigsted et al. [17] identified altered landing mechanics despite comparable hop distance, while Kotsifaki et al. [18] showed that unilateral vertical jumps may reveal knee-function differences that are not apparent from horizontal hop distance alone. Read et al. [24] reported distinct asymmetry profiles during eccentric, propulsive, and landing phases in professional football players after ACLR. The present results add to this evidence by showing that the greatest individual differences were not concentrated in a single global performance measure but appeared in specific phases and directions. However, without preinjury data or a control group, these asymmetries cannot be attributed solely to ACLR and should instead be interpreted within each player’s broader functional profile.

The largest asymmetries occurred during the most complex task, which combined landing with immediate re-propulsion. During the unilateral forward hop onto the platform followed by immediate take-off and landing beyond the platform, median asymmetry was 40.8% for AP braking impulse and 23.4% for AP re-propulsive impulse. Substantial differences were also observed in stabilization time: median asymmetry was 26.4% in the AP direction following the unilateral forward hop onto the platform and 20.7% in the ML direction following the unilateral lateral hop onto the platform. The greatest inter-limb differences therefore occurred when players were required to rapidly attenuate momentum, redirect force, and regain control after contact rather than during isolated maximal torque production. The task-specific nature of these findings is consistent with the previous literature. Markström et al. [26] showed that side hops imposed greater frontal- and transverse-plane control demands than vertical or forward hops. Head et al. [27] and Wikstrom et al. [28] demonstrated that landing direction modifies stability demands in the vertical, AP, and ML directions. Time to stabilization is a particularly complex outcome because it reflects the attenuation of post-landing oscillations through sensory information and the coordinated contribution of the ankle, knee, hip, and trunk. Webster and Gribble [29] reported prolonged time to stabilization in female athletes approximately 2.5 years after ACLR, and Niederer et al. [30] showed that altered landing control may remain evident after return to activity. The weak relationship between stabilization time and isokinetic strength is therefore plausible, as knee strength may be only one of several factors associated with post-landing control; however, the present results do not establish its independent contribution. The relatively large percentage asymmetries observed for braking and stabilization should also be interpreted cautiously. Percentage asymmetry may increase when the underlying values are small, while the reliability and typical variability of impulse and TTS measures may differ from those of peak torque or jump height. These findings therefore describe inter-limb differences within the present sample but do not establish that they exceeded measurement error.

Individual-level analysis further showed that small asymmetries in knee strength and primary vertical jump outcomes did not exclude larger differences during directional tasks. Using the 20% threshold, every player demonstrated at least one braking, re-propulsion, or stabilization variable above this value. Because an isolated threshold exceedance may occur by chance, the typical magnitude of asymmetry across the complete directional-task set was also considered. In half of the players, the median directional-task asymmetry was at least 20%, indicating that the differences in these individuals were not restricted to a single isolated variable. Importantly, half of the players whose knee strength and two primary vertical outcomes remained below the 20% asymmetry threshold still demonstrated a median directional-task asymmetry of at least 20%. Asymmetries exceeding 20% occurred more frequently exclusively during directional tasks than exclusively in strength and primary vertical outcomes (Holm-adjusted *p* = 0.016). This descriptive difference remained statistically significant when sets containing an equal number of variables were compared (Holm-adjusted *p* = 0.016), suggesting that the observed pattern was not solely attributable to the larger number of directional outcomes. However, matching the number of outcomes does not equalize their reliability, typical variability, or clinical meaning. The threshold-based findings should be interpreted descriptively. The 20% criterion was intended to identify clearly larger individual inter-limb differences but has not been validated for the directional force-impulse or stabilization measures examined. Therefore, exceeding this threshold should not be interpreted as evidence of clinical abnormality a clinically validated impairment, or diagnostic status.

During bilateral tasks, the operated limb contributed an average of 49.9–53.0% of the analyzed impulses, and its contribution during the CMJ and DJ was also close to half of the total outcome. No consistent group-level pattern of operated-limb unloading was therefore identified. Greater strength asymmetry was not systematically associated with a smaller operated-limb contribution, and strength–performance relationships were not demonstrably stronger during unilateral tasks. These findings do not exclude compensation in individual players. Bilateral tasks allow work to be redistributed between limbs [24,31,32], whereas unilateral tasks restrict direct transfer to the contralateral side but increase the demands placed on balance, technique, and pelvic and trunk control [33,34]. The two conditions consequently provide different information: bilateral tasks characterize the distribution of work between limbs, while unilateral tasks assess the capacity of each limb to accept load, generate propulsion, and stabilize independently.

From a practical perspective, the findings suggest the potential value of assessment at several complementary levels. Isokinetic dynamometry quantifies the isolated torque-generating capacity of the knee extensors and flexors. The SLCMJ indicates how this capacity is used during a single vertical propulsion, whereas the SLDJ assesses rapid transition from load acceptance to renewed propulsion. Forward and lateral tasks extend the assessment to directional braking, re-acceleration, and the recovery of stability. No single measurement should replace the others because each addresses a different component of the player’s functional profile. Lower strength accompanied by lower SLCMJ height or propulsive impulse may indicate lower values in both muscular capacity and its dynamic expression. Relatively similar isokinetic strength values accompanied by marked braking or stabilization asymmetry may warrant closer assessment of load acceptance, rapid transition to re-propulsion, and AP and ML control. Conversely, a favorable jump outcome in the presence of lower strength may reflect different contributions from other joints and should not be used as the sole basis for discontinuing strength development. Even favorable results across all domains cannot independently confirm complete functional recovery in the absence of preinjury data and an appropriate external reference. The purpose of this test battery is not to generate a single composite score or to count the number of passed thresholds. Its value lies in identifying an individual profile that includes the absolute capacity of each limb, the magnitude and direction of asymmetry, the movement phase in which a difference occurs, and the intensity with which the task was performed. Assessment should be repeated over time, and observed changes should be interpreted in relation to measurement reliability, positional demands, and current training and match exposure [8,40]. The practical application of these findings does not require every club to maintain a high-frequency tri-axial force-platform system. When such equipment is unavailable, periodic referral to a biomechanics or sports-science laboratory may be considered at key rehabilitation or RTS stages. In routine club practice, standardized forward and lateral single-leg hop-and-stabilization tasks can be assessed using hop distance, observable stabilization errors, and frontal- or sagittal-plane video. These field-based approaches do not reproduce AP and ML impulse measurements but may provide complementary information about directional braking and landing control and help indicate when more detailed laboratory assessment may be useful.

Several limitations should be acknowledged. The small sample and variable data availability reduced statistical power, precision, and generalizability; therefore, non-significant results do not establish limb equivalence or the absence of smaller associations. The cross-sectional design, absence of preinjury data and a healthy control group, and heterogeneous time since ACLR (12–40 months) prevent causal interpretation and determination of whether the observed asymmetries were abnormal or attributable to ACLR. The findings may also have been influenced by differences in rehabilitation and sport exposure. The fixed test order and self-selected approach intensity and hop distance may have influenced task performance. Because only external ground reaction forces were measured, joint kinematics, joint moments, internal loading, and ACL strain could not be determined. Reliability and measurement error were not independently assessed, and the 20% asymmetry threshold was an exploratory descriptive criterion rather than a validated clinical cut-off. Selection bias is possible because the sample included only professional players who had completed the RTS process and returned to unrestricted training and competition. In addition, vertical jump and directional tasks were recorded using two different force-plate systems, which may limit direct comparability across measurement domains.

## 5. Conclusions

The main practical implication of this study is that isokinetic strength results alone do not fully characterize how a player accepts load, re-propels the body, or regains stability during directional tasks. Isokinetic dynamometry quantifies torque production under controlled conditions but does not show how a player accepts load, decelerates the body, re-propels, or regains stability during movement in different directions. In the present group, the absence of significant operated–non-operated differences in strength and vertical jump outcomes coexisted with larger inter-limb differences in selected directional braking and post-landing stabilization measures. An assessment limited to isokinetic strength and basic vertical tests may not fully characterize individual performance during directional braking, re-acceleration, and post-landing stabilization. Unilateral forward and lateral tasks may therefore provide complementary information about components of performance not captured by strength and vertical jump outcomes alone. Isokinetic dynamometry, vertical jumps, and multidirectional tasks may be considered complementary rather than interchangeable components of assessment. Their combined interpretation may provide a more complete description of an individual functional profile; however, the present findings do not establish causality, clinical abnormality, persistent consequences of ACLR, diagnostic accuracy, or clinical thresholds and require confirmation in larger samples. Where multi-planar force platforms are unavailable, standardized forward and lateral single-leg landing tasks supported by two-dimensional video may provide practical complementary information, while targeted laboratory assessment may be considered at key rehabilitation or RTS stages.

## Figures and Tables

**Table 1:** Comparison of isokinetic strength, vertical jump, and directional task outcomes between the operated and non-operated limbs.

Outcome	*n*	Operated Limb	Non-Operated Limb	Paired Difference O−NO [95% CI]	Effect Size	*p*	*p*_Holm_	Median |A| (%)
Knee extensors PT/BW at 60°/s	11	2.915 ± 0.566	3.040 ± 0.375	−0.125 [−0.355, 0.105]	*g*z = −0.338	0.252	0.723	13.4
Knee flexors PT/BW at 60°/s	11	1.704 ± 0.295	1.841 ± 0.219	−0.137 [−0.301, 0.027]	*g*z = −0.520	0.091	0.549	7.8
SLCMJ height	10	0.184 ± 0.045	0.178 ± 0.042	0.006 [−0.011, 0.024]	*g*z = 0.237	0.434	1.000	8.4
SLDJ RSI	10	1.311 ± 0.387	1.283 ± 0.412	0.028 [−0.082, 0.138]	*g*z = 0.167	0.578	1.000	9.8
Forward hop-in AP braking impulse	10	1.361 ± 0.383	1.332 ± 0.226	0.029 [−0.161, 0.220]	*g*z = 0.101	0.736	1.000	14.4
Forward hop-out AP propulsive impulse	9	1.663 ± 0.450	1.703 ± 0.461	−0.040 [−0.117, 0.036]	*g*z = −0.367	0.258	1.000	3.7
Forward hop-in–out AP braking impulse	10	0.231 ± 0.173	0.220 ± 0.103	0.011 [−0.098, 0.121]	*g*z = 0.068	0.820	1.000	40.8
Forward hop-in–out AP re-propulsive impulse	10	0.363 ± 0.140	0.276 ± 0.178	0.087 [−0.005, 0.180]	*g*z = 0.618	0.061	0.306	23.4
Forward hop-in–out contact time	10	0.300 [0.274–0.379]	0.284 [0.244–0.334]	0.028 [0.009, 0.048]	*r*rb = 0.745	0.037	0.223	9.9
Lateral hop-in ML braking impulse	10	1.087 ± 0.233	1.055 ± 0.199	0.032 [−0.109, 0.174]	*g*z = 0.150	0.617	1.000	12.5
Lateral hop-in ML TTS	10	0.983 ± 0.365	1.019 ± 0.327	−0.036 [−0.238, 0.166]	*g*z = −0.117	0.740	1.000	20.7
Lateral hop-out ML propulsive impulse	11	1.348 [1.249–1.478]	1.359 [1.162–1.500]	0.019 [−0.038, 0.124]	*r*rb = 0.121	0.765	1.000	3.4

Data are presented as mean ± SD or median [Q1–Q3], according to the distribution of paired differences and the statistical test applied. The paired difference is reported as the mean difference for paired-samples *t*-tests and as the median difference for Wilcoxon signed-rank tests, with bootstrap 95% confidence intervals. Positive values indicate higher results for the operated limb. Effect sizes are reported as Hedges’ *g*z for paired-samples *t*-tests and rank-biserial correlation (*r*rb) for Wilcoxon tests. Holm adjustments were applied separately to the following families: isokinetic PT/BW outcomes at 60°/s, 180°/s, and 300°/s (six comparisons); vertical jump outcomes (seven comparisons); forward directional-task outcomes (six comparisons); and lateral directional-task outcomes (three comparisons). The value of *n* represents the number of participants with a valid paired result for the respective outcome; missing observations were not imputed. PT/BW, peak torque normalized to body mass; SLCMJ, single-leg countermovement jump; SLDJ, single-leg drop jump; RSI, reactive strength index; AP, anterior–posterior; ML, medial–lateral; TTS, time to stabilization; |A|, absolute interlimb asymmetry; SD, standard deviation; Q1–Q3, first and third quartiles. PT/BW is expressed in Nm/kg, jump height in m, RSI in m·s^−1^, impulses in N·s·kg^−1^, and contact time and TTS in s. *p*, unadjusted probability value; *p*_Holm_, probability value adjusted using the Holm method.

**Table 2:** Correlations between operated-limb isokinetic strength and dynamic task outcomes.

Strength Variable	Dynamic Task Outcome	Spearman ρ	95% CI	*p*	*p*_Holm_
Knee extensors	SLCMJ height	0.491	−0.246 to 0.923	0.158	0.474
Knee extensors	SLDJ RSI	0.612	−0.259 to 0.963	0.068	0.271
Knee extensors	Forward hop-in AP braking impulse	0.382	−0.409 to 0.849	0.279	0.474
Knee extensors	Forward hop-out AP propulsive impulse	0.442	−0.387 to 0.860	0.206	0.474
Knee flexors	SLCMJ height	0.152	−0.597 to 0.691	0.680	1.000
Knee flexors	SLDJ RSI	0.188	−0.635 to 0.648	0.607	1.000
Knee flexors	Forward hop-in AP braking impulse	0.515	−0.312 to 0.987	0.134	0.537
Knee flexors	Forward hop-out AP propulsive impulse	0.382	−0.388 to 0.819	0.283	0.849

Abbreviations: SLCMJ—single-leg countermovement jump; SLDJ—single-leg drop jump; RSI—reactive strength index; AP—anterior–posterior direction; ρ—Spearman rank correlation coefficient; CI—confidence interval; *p*—unadjusted probability value obtained using a Monte Carlo permutation procedure; Holm-adjusted *p*—probability value adjusted using the Holm method. Confidence intervals were calculated using the bias-corrected and accelerated (BCa) bootstrap. A positive ρ indicates that greater strength tended to accompany a higher value of the analyzed outcome.

**Table 3:** Absolute inter-limb asymmetry across functional domains.

Functional Domain	Median (%)	Q1–Q3 (%)
Isokinetic knee extensor and flexor strength	9.9	8.5–12.3
Vertical jump outcomes	6.2	4.5–8.5
Braking during directional tasks	14.7	13.9–15.3
Propulsion and re-acceleration	7.3	4.6–12.4
Temporal control and stabilization	20.9	16.4–26.7

For each player, absolute asymmetry was summarized as the median within each domain. Values are presented as the median [Q1–Q3] of these player-level domain medians. Q1–Q3, first and third quartiles. Absolute asymmetry describes the magnitude of the inter-limb difference irrespective of direction.

**Table 4:** Absolute inter-limb asymmetry and frequency of values at or above 20% for selected strength, vertical jump, and directional task outcomes.

Outcome	Median |A| (%)	*n*/*N* (%) ≥ 20%
Knee extensors PT/BW at 60°/s	13.4	0/11 (0.0)
Knee flexors PT/BW at 60°/s	7.8	2/11 (18.2)
SLCMJ height	8.4	1/10 (10.0)
SLDJ height	12.1	2/10 (20.0)
Forward hop-in–out AP braking impulse	40.8	7/10 (70.0)
Forward hop-in–out AP re-propulsive impulse	23.4	5/10 (50.0)
Forward hop-in AP TTS	26.4	6/9 (66.7)
Lateral hop-in ML TTS	20.7	5/10 (50.0)

PT/BW—peak torque normalized to body mass; SLCMJ—single-leg countermovement jump; SLDJ—single-leg drop jump; AP—anterior–posterior direction; ML—medial–lateral direction; TTS—time to stabilization; |A|—absolute interlimb asymmetry. The directional set comprised forward hop-in AP braking impulse and AP TTS; forward hop-out AP propulsive impulse; forward hop-in–out AP braking impulse, AP re-propulsive impulse, and contact time; lateral hop-in ML braking impulse and ML TTS; and lateral hop-out ML propulsive impulse. Values are presented as *n*/*N* (%), where *n* is the number of players meeting the 20% threshold and N is the number of players with valid data for the respective outcome.

**Table 5:** Contribution of the operated limb to bilateral directional task outcomes.

Outcome	Mean Operated-Limb Contribution (%)	SD (%)	vs. 50%: *p*_Holm_
Bilateral forward hop-in AP braking	53.0	5.9	0.465
Bilateral forward hop-out AP propulsion	49.9	4.6	1.000
Bilateral forward hop-in–out AP braking	50.2	7.5	1.000
Bilateral forward hop-in–out AP re-propulsion	51.7	7.4	1.000

Abbreviations: AP—anterior–posterior direction; SD—standard deviation; Holm-adjusted *p*—probability value for comparison with 50%, adjusted using the Holm method. A contribution of 50% represents equal contributions from the operated and non-operated limbs to the combined task outcome.

## Data Availability

The individual-level data supporting the findings of this study are available from the corresponding author on reasonable request.

## Appendix A

### Appendix A.1

**Table A1:** Isokinetic knee-strength outcomes at 180°/s and 300°/s.

Velocity	Muscle Group	*n*	Operated Limb	Non-Operated Limb	O−NO [95% CI]	*p*	*p*_Holm_	Median |A| (%)
180°/s	KE	11	1.917 ± 0.451	1.967 ± 0.346	−0.050 [−0.208, 0.108]	0.499	0.723	8.4
180°/s	KF	11	1.311 ± 0.192	1.363 ± 0.205	−0.052 [−0.142, 0.038]	0.229	0.723	10.2
300°/s	KE	11	1.410 ± 0.198	1.363 ± 0.193	−0.090 [−0.146, 0.013]	0.102	0.549	7.8
300°/s	KF	11	0.993 ± 0.198	1.054 ± 0.192	−0.062 [−0.158, 0.034]	0.181	0.723	12.7

Values are presented as mean ± standard deviation (SD) for outcomes analyzed using the paired-samples *t*-test and as median [Q1–Q3] for outcomes analyzed using the Wilcoxon signed-rank test. O−NO represents the paired difference between the operated and non-operated limbs; positive values indicate higher values for the operated limb. Differences are presented with bootstrap 95% confidence intervals calculated using the bias-corrected and accelerated (BCa) method. The value of *n* represents the number of participants with valid paired measurements. The *p*-value is the unadjusted probability value obtained using the paired-samples *t*-test or Wilcoxon signed-rank test, as appropriate. Holm-adjusted *p* represents the probability value adjusted within the prespecified family of six knee-extensor and knee-flexor PT/BW comparisons performed at 60°/s, 180°/s, and 300°/s. Abbreviations: PT/BW—peak torque normalized to body mass; O—operated limb; NO—non-operated limb; CI—confidence interval; Q1–Q3—first and third quartiles; |A|—absolute interlimb asymmetry. PT/BW is expressed in Nm/kg. For paired-samples t-tests, O−NO is the mean paired difference; for Wilcoxon signed-rank tests, it is the median paired difference.

### Appendix A.2

**Table A2:** I Associations between operated-limb knee strength at 180°/s and 300°/s and dynamic-task outcomes.

Velocity	Muscle	Fam.	Dynamic Outcome	*n*	ρ	95% CI	*p*	*p*_Holm_
180°/s	KE	P	SLCMJ height	10	0.491	[−0.231, 0.922]	0.160	0.480
180°/s	KE	A	SLCMJ net propulsive impulse	10	0.491	[−0.231, 0.922]	0.157	1.000
180°/s	KE	P	SLDJ RSI	10	0.612	[−0.199, 0.987]	0.064	0.257
180°/s	KE	A	SLDJ contact time	10	−0.491	[−0.938, 0.412]	0.156	1.000
180°/s	KE	A	SLDJ height	10	0.394	[−0.362, 0.714]	0.268	1.000
180°/s	KE	A	SLDJ net braking impulse	10	0.455	[−0.180, 0.883]	0.190	1.000
180°/s	KE	A	SLDJ net propulsive impulse	10	0.394	[−0.362, 0.716]	0.265	1.000
180°/s	KE	P	Forward hop-in AP braking impulse	10	0.430	[−0.379, 0.875]	0.212	0.480
180°/s	KE	A	Forward hop-in AP TTS	9	−0.143	[−0.882, 1.000]	0.783	1.000
180°/s	KE	P	Forward hop-out AP propulsive impulse	9	0.394	[−0.468, 0.875]	0.260	0.480
180°/s	KE	A	Forward hop-in–out AP braking impulse	10	−0.479	[−0.822, 0.212]	0.165	1.000
180°/s	KE	A	Forward hop-in–out AP re-propulsive impulse	10	−0.188	[−0.823, 0.700]	0.608	1.000
180°/s	KE	A	Forward hop-in–out contact time	10	−0.624	[−0.948, −0.094]	0.059	0.707
180°/s	KE	A	Lateral hop-in ML braking impulse	10	0.355	[−0.393, 0.766]	0.286	1.000
180°/s	KE	A	Lateral hop-in ML TTS	10	0.107	[−0.703, 0.862]	0.765	1.000
180°/s	KE	A	Lateral hop-out ML propulsive impulse	11	0.236	[−0.521, 0.729]	0.485	1.000
180°/s	KF	P	SLCMJ height	10	0.079	[−0.602, 0.700]	0.841	1.000
180°/s	KF	A	SLCMJ net propulsive impulse	10	0.079	[−0.597, 0.692]	0.834	1.000
180°/s	KF	P	SLDJ RSI	10	−0.006	[−0.660, 0.627]	1.000	1.000
180°/s	KF	A	SLDJ contact time	10	0.079	[−0.623, 0.679]	0.843	1.000
180°/s	KF	A	SLDJ height	10	−0.188	[−0.802, 0.519]	0.609	1.000
180°/s	KF	A	SLDJ net braking impulse	10	0.261	[−0.538, 0.774]	0.468	1.000
180°/s	KF	A	SLDJ net propulsive impulse	10	−0.188	[−0.808, 0.519]	0.615	1.000
180°/s	KF	P	Forward hop-in AP braking impulse	10	0.273	[−0.457, 0.889]	0.459	1.000
180°/s	KF	A	Forward hop-in AP TTS	9	0.179	[−0.882, 0.961]	0.713	1.000
180°/s	KF	P	Forward hop-out AP propulsive impulse	9	0.018	[−0.543, 0.801]	0.973	1.000
180°/s	KF	A	Forward hop-in–out AP braking impulse	10	0.188	[−0.509, 0.709]	0.605	1.000
180°/s	KF	A	Forward hop-in–out AP re-propulsive impulse	10	0.115	[−0.750, 0.819]	0.754	1.000
180°/s	KF	A	Forward hop-in–out contact time	10	−0.200	[−0.847, 0.623]	0.582	1.000
180°/s	KF	A	Lateral hop-in ML braking impulse	10	0.473	[−0.212, 0.906]	0.145	1.000
180°/s	KF	A	Lateral hop-in ML TTS	10	0.192	[−0.581, 0.752]	0.596	1.000
180°/s	KF	A	Lateral hop-out ML propulsive impulse	11	0.073	[−0.593, 0.692]	0.838	1.000
300°/s	KE	P	SLCMJ height	10	0.479	[−0.247, 0.873]	0.166	0.343
300°/s	KE	A	SLCMJ net propulsive impulse	10	0.479	[−0.233, 0.868]	0.168	1.000
300°/s	KE	P	SLDJ RSI	10	0.673	[0.044, 0.963]	0.039	0.154
300°/s	KE	A	SLDJ contact time	10	−0.552	[−0.925, 0.218]	0.107	1.000
300°/s	KE	A	SLDJ height	10	0.394	[−0.373, 0.698]	0.261	1.000
300°/s	KE	A	SLDJ net braking impulse	10	0.503	[−0.082, 0.885]	0.145	1.000
300°/s	KE	A	SLDJ net propulsive impulse	10	0.394	[−0.400, 0.689]	0.263	1.000
300°/s	KE	P	Forward hop-in AP braking impulse	10	0.527	[−0.176, 0.925]	0.122	0.343
300°/s	KE	A	Forward hop-in AP TTS	9	−0.321	[−1.000, 0.882]	0.498	1.000
300°/s	KE	P	Forward hop-out AP propulsive impulse	9	0.539	[−0.245, 0.925]	0.114	0.343
300°/s	KE	A	Forward hop-in–out AP braking impulse	10	−0.576	[−0.875, 0.247]	0.090	0.991
300°/s	KE	A	Forward hop-in–out AP re-propulsive impulse	10	−0.127	[−0.887, 0.806]	0.728	1.000
300°/s	KE	A	Forward hop-in–out contact time	10	−0.770	[−0.987, −0.221]	0.013	0.155
300°/s	KE	A	Lateral hop-in ML braking impulse	10	0.400	[−0.324, 0.850]	0.225	1.000
300°/s	KE	A	Lateral hop-in ML TTS	10	0.034	[−0.805, 0.682]	0.923	1.000
300°/s	KE	A	Lateral hop-out ML propulsive impulse	11	0.373	[−0.384, 0.722]	0.257	1.000
300°/s	KF	P	SLCMJ height	10	0.394	[−0.615, 0.688]	0.258	0.258
300°/s	KF	A	SLCMJ net propulsive impulse	10	0.394	[−0.590, 0.698]	0.261	1.000
300°/s	KF	P	SLDJ RSI	10	0.576	[−0.346, 0.925]	0.088	0.241
300°/s	KF	A	SLDJ contact time	10	−0.479	[−0.963, 0.456]	0.165	1.000
300°/s	KF	A	SLDJ height	10	0.200	[−0.620, 0.714]	0.579	1.000
300°/s	KF	A	SLDJ net braking impulse	10	0.552	[−0.077, 0.844]	0.105	1.000
300°/s	KF	A	SLDJ net propulsive impulse	10	0.200	[−0.610, 0.711]	0.587	1.000
300°/s	KF	P	Forward hop-in AP braking impulse	10	0.648	[−0.200, 0.987]	0.049	0.194
300°/s	KF	A	Forward hop-in AP TTS	9	−0.179	[−0.887, 0.765]	0.713	1.000
300°/s	KF	P	Forward hop-out AP propulsive impulse	9	0.588	[−0.156, 0.887]	0.080	0.241
300°/s	KF	A	Forward hop-in–out AP braking impulse	10	−0.491	[−0.883, 0.138]	0.157	1.000
300°/s	KF	A	Forward hop-in–out AP re-propulsive impulse	10	0.042	[−0.692, 0.790]	0.916	1.000
300°/s	KF	A	Forward hop-in–out contact time	10	−0.721	[−0.963, 0.016]	0.023	0.250
300°/s	KF	A	Lateral hop-in ML braking impulse	10	0.791	[−0.047, 1.000]	0.005	0.064
300°/s	KF	A	Lateral hop-in ML TTS	10	0.143	[−0.748, 0.787]	0.690	1.000
300°/s	KF	A	Lateral hop-out ML propulsive impulse	11	0.464	[−0.217, 0.794]	0.153	1.000

Associations were calculated between operated-limb peak torque normalized to body mass at the specified angular velocity and the corresponding dynamic-task outcome from the same limb. P comprised the four prespecified dynamic outcomes: SLCMJ height, SLDJ RSI, forward hop-in AP braking impulse, and forward hop-out AP propulsive impulse; A comprised the remaining exploratory outcomes. Missing observations were not imputed. Holm adjustments were applied separately within each combination of testing velocity, muscle group, and analysis family. Confidence intervals were calculated using the bias-corrected and accelerated (BCa) bootstrap. A positive ρ indicates that greater strength tended to accompany a higher value of the analyzed outcome. Abbreviations: KE—knee extensors; KF—knee flexors; P—primary; A—additional; SLCMJ—single-leg countermovement jump; SLDJ—single-leg drop jump; RSI—reactive strength index; AP—anterior–posterior; ML—medial–lateral; TTS—time to stabilization; ρ—Spearman rank correlation coefficient; CI—confidence interval; *p*—unadjusted probability value obtained using a Monte Carlo permutation procedure; *p*_Holm_—probability value adjusted using the Holm method.

### Appendix A.3

**Table A3:** Comparison of strength–performance correlations between corresponding unilateral and bilateral directional tasks.

Corresponding Task and Outcome	*n*	ρ Unilateral	ρ Bilateral	Δρ Uni−Bilateral	Bootstrap 95% CI	*p*Bootstrap
Forward hop-in AP braking impulse	10	0.382	0.370	0.012	[−0.359, 0.469]	0.945
Forward hop-out AP propulsive impulse	10	0.442	0.430	0.012	[−0.497, 0.494]	0.874
Forward hop-in–out AP braking impulse	10	−0.576	−0.636	0.061	[−0.428, 0.617]	0.863
Forward hop-in–out AP re-propulsive impulse	10	−0.236	0.018	−0.255	[−1.070, 0.633]	0.546

The table compares Spearman correlations between operated-limb knee-extensor PT/BW at 60°/s and corresponding outcomes obtained during unilateral and bilateral variants of the same task. The difference between correlations was calculated as Δρ = ρ_unilateral − ρ_bilateral. Positive values indicate a stronger positive association for the unilateral task, whereas negative values indicate a stronger association for the bilateral task. Bootstrap 95% confidence intervals and two-sided *p* values were calculated using participant-level paired resampling, with the same resampled players contributing to both correlations. Abbreviations: AP—anterior–posterior direction; PT/BW—peak torque normalized to body mass; ρ—Spearman rank correlation coefficient; Δρ—difference between correlation coefficients; CI—confidence interval; *p* bootstrap—probability value obtained from the paired-bootstrap comparison. Sample sizes may differ from Table 1 because the paired limb comparisons required valid outcomes for both limbs, whereas these correlation analyses required an operated-limb outcome and the corresponding strength value.
